# Left, right, left, right, eyes to the front! Müller-Lyer bias in grasping is not a function of hand used, hand preferred or visual hemifield, but foveation does matter

**DOI:** 10.1007/s00221-012-3007-x

**Published:** 2012-01-26

**Authors:** John van der Kamp, Matthieu M. de Wit, Rich S. W. Masters

**Affiliations:** 1 Institute of Human Performance, University of Hong Kong, Pokfulam, Hong Kong; 2 Research Institute MOVE, Faculty of Human Movement Sciences, VU University Amsterdam, Van der Boechorststraat 9, 1081 BT Amsterdam, The Netherlands

**Keywords:** Visual illusions, Handedness, Perception–action, Visual hemifield, Peripheral visual field

## Abstract

We investigated whether the control of movement of the left hand is more likely to involve the use of allocentric information than movements performed with the right hand. Previous studies (Gonzalez et al. in J Neurophys 95:3496–3501, 2006; De Grave et al. in Exp Br Res 193:421–427, 2009) have reported contradictory findings in this respect. In the present study, right-handed participants (*N* = 12) and left-handed participants (*N* = 12) made right- and left-handed grasps to foveated objects and peripheral, non-foveated objects that were located in the right or left visual hemifield and embedded within a Müller-Lyer illusion. They were also asked to judge the size of the object by matching their hand aperture to its length. Hand apertures did not show significant differences in illusory bias as a function of hand used, handedness or visual hemifield. However, the illusory effect was significantly larger for perception than for action, and for the non-foveated compared to foveated objects. No significant illusory biases were found for reach movement times. These findings are consistent with the two-visual system model that holds that the use of allocentric information is more prominent in perception than in movement control. We propose that the increased involvement of allocentric information in movements toward peripheral, non-foveated objects may be a consequence of more awkward, less automatized grasps of nonfoveated than foveated objects. The current study does not support the conjecture that the control of left-handed and right-handed grasps is predicated on different sources of information.

## Introduction

Recently, a possible disparity in the degree to which the right and left hand are susceptible to optical illusions when grasping or pointing at objects has received considerable attention (De Grave et al. 2009; Gonzalez et al. 2006; Radoeva et al. 2005). The interest is motivated by the two-visual systems model for action and perception, which proposes that movement control and perception are dissociated, not only in relation to the underlying neuro-anatomical separation of the dorsal and ventral pathways, but also with respect to the visual information that they rely upon (Milner and Goodale 1995, 2008; de Wit et al. 2011). The empirical support for this dissociation is partly grounded in intriguing but controversial observations that the perception of objects is much more affected by illusory configurations (and hence visual context) than movements directed toward those objects (Bruno et al. 2008; Ganel et al. 2008; cf. Franz et al. 2009; Smeets and Brenner 2006). This neatly concurs with the functional demands of perception and action. Perception gathers knowledge about the environment predicated on the use of allocentric information that specifies objects and their properties relative to other objects of the environment. Action controls the movements that are directed to the objects by exploiting egocentric information that specifies the object relative to the body. The information-based distinction, however, also implies that an illusory bias in object-directed movements (however small) points to the exploitation of both egocentric and allocentric information for movement control, indicating that the visual systems for perception and action normally do not operate in total isolation.

In this regard, Gonzalez et al. (2006) reported that hand aperture during grasping with the left hand, but not with the right hand, was affected by Ponzo and Ebbinghaus size illusions. This asymmetry in illusory bias between right- and left-handed grasping was found in right-handed as well as in left-handed participants, indicating that the difference occurred irrespective of handedness. These observations are pertinent in that they may suggest another instance of interactive contributions from the two visual systems in movement control. Yet, the empirical evidence for an asymmetrical illusory bias between the right- and left-hand is contentious. For example, Gentilucci et al. (1997) reported that the bias in pointing movements directed towards the vertex of Müller-Lyer configurations was the same for one sample of participants who pointed with their right hand and a second sample of participants who pointed with their left hand. Likewise, de Grave et al. (2009), using a within-participant comparison, found that the Brentano illusion affected the accuracy of pointing movements of right-handers with their right and left hand to the same extent. Yet, in a study involving patients with unilateral brain damage, Radoeva et al. (2005) found that patients with left-hemisphere damage who grasped shafts embedded in Müller-Lyer configurations with their (unaffected) left hand had a larger illusory bias in hand aperture than patients with right-hemisphere damage who grasped with their right hand. However, because right-handed grasps were always directed to the (unaffected) left visual hemifield and vice versa, the different illusory influences for the two hands can also be attributed to the visual hemifield in which the object was presented. In addition, no illusion-related differences in hand aperture between the right and left hand were found in a group of right-handed control participants. Finally, Adam et al. (2010) recently reported that aiming movements with the left hand were more sensitive to visual context than movements with the right hand. Rather than employing the illusion paradigm, these authors built on earlier observations that the presence of a linear array of placeholders (one of which is the target) violated the relationship between movement time and object distance (i.e., Fitts’s Law; Adam et al. 2006). This effect of visual context (i.e., placeholders) on movement time suggests involvement of allocentric sources of information for controlling reaching movements. Adam et al. (2010) showed that the violation of Fitts’s Law was greater for reaches with the left hand. These discrepancies in both the findings and methodology of previous work warrant further scrutiny.

In order to account for an asymmetrical illusory bias for the right and left hands two explanations need to be distinguished. First, the visuomotor networks for action in the dorsal pathway may be more strongly dissociated from the networks for perception in the ventral pathway in the left hemisphere than in the right hemisphere. That is, the encapsulated visuomotor networks for action in the dorsal pathway may have evolved preferentially in the left hemisphere (Gonzalez et al. 2006; Perenin and Vighetto 1988), perhaps as a consequence of human language taking up or co-opting networks in the left hemisphere located between the dorsal and ventral pathways, whereas in the right hemisphere these networks were retained for visual perception (Radoeva et al. 2005; Corballis et al. 2000). All the same, a stronger dissociation between the two pathways in the left hemisphere may limit their interaction, leaving right-handed movements less prone to the use of allocentric illusion-inducing information than movements with the left hand. The evolutionary argument should be distinguished from an experience-dependent explanation concerning the interactive contributions of the two visual systems. Gonzalez et al. (2008), for example, found that unfamiliar awkward grips were much more susceptible to a size-contrast illusion than the precision grips that participants habitually used to grasp small objects. This finding points toward new or less-practiced actions being more reliant on allocentric information than well-practiced automatized actions, perhaps suggesting less encapsulation of the dorsal pathway in early stages of motor learning (Gonzalez et al. 2008; van der Kamp et al. 2003). Consequently, if the asymmetry in illusory bias is experience-dependent then its *direction* should depend on handedness: in participants with right hand preference the illusory bias should be larger in the less practiced grasps with the left hand, while participants with a left hand preference should show a larger bias when grasping with their right hand (cf. Gonzalez et al. 2006).

The possibility that the observed asymmetry in illusory bias between the right and left hand in fact results from the visual hemifield in which the object is presented, should also be considered. On the same evolutionary perspective (Corballis et al. 2000), networks for visual perception occupy more cortical space in the right hemisphere as compared to the left hemisphere. And also Milner and Goodale (1995, p. 112, pp. 150–151) originally argued that hemispheric asymmetry is much more apparent for ventral than for dorsal pathways. Indeed, perception research suggests that susceptibility to illusions is higher when they are presented to the left visual field as compared to the right visual hemifield (e.g., Clem and Pollack 1975; Rasmjou et al. 1999), although the evidence is not unambiguous (e.g., Bertelson and Morais 1983). It is noticeable therefore that the studies that did report an asymmetrical bias for right-handed and left-handed grasping did not systematically control for the visual hemifield in which the illusory object was presented (Gonzalez et al. 2006; Radoeva et al. 2005, see also Adam et al. 2010). Hence, it cannot be ruled out that the different illusion effects for right-handed and left-handed grasping simply reflect differences in visual perception depending on visual hemifield rather than hand use. In the study by Radoeva et al. (2005), for example, participants with damage to the right hemisphere only grasped with their right hand to objects embedded in a Müller-Lyer configuration that were presented in the right visual hemifield, and conversely, participants with left hemispheric damage solely grasped with their left hand to objects in the left visual hemifield. The latter group showed a larger illusory bias. The same holds for the study by Adam et al. (2010). Reaching movements with the left hand were only made to targets presented in the left visual hemifield, while right-handed reaches were only made to targets presented in the right visual hemifield. Reaches with the left hand to targets in the left hemifield appeared more vulnerable to visual context. Also Gentilucci et al. (1997) reported that participants tended to show a somewhat larger illusory bias when pointing to the vertex of Müller-Lyer configurations located in the left visual field. By contrast, the between-participants comparison for right-handed and left-handed pointing movements did not reveal an asymmetrical illusory bias.

In the study that we report, we asked participants in an action task to reach and grasp a shaft embedded in a Müller-Lyer figure with either their right or left hand. Additionally, we asked them in a perception task to match hand aperture to the length of the shaft, again using their right or left hand. We reasoned that the observation of an illusory bias would point to the exploitation of allocentric or contextual information, which presumably reflects contributions of the visual perception system in the ventral pathway. We expected a more reliable illusory effect to emerge for the grasp (i.e., hand aperture) compared to the reach (i.e., reaching movement time), because a Müller-Lyer figure induces an illusion of size and not of distance. However, given previous reports that allocentric information may be differentially involved in reaches performed by the left and right hand (Adam et al. 2010), we also assessed the illusory bias for the reach. We specifically tested the proposition that the visuomotor networks in the dorsal pathway of the left hemisphere are more encapsulated (i.e., more dissociated from the visual perception networks in the ventral pathway) than those in the right hemisphere. If correct, then an asymmetry in illusory bias in grasping (and perhaps reaching) should occur with the bias being larger for left-handed movements than for right-handed movements (i.e., showing greater reliance on allocentric information in left-handed movements). We explicitly aimed to disentangle any asymmetrical bias between the right and left hands from influences of handedness (or hand preference) and visual hemifield. Hence, right-handed as well as left-handed participants performed both the action and perception task with the shaft presented in the centre of the visual field, in the right visual hemifield (i.e., participants fixated to the left of the shaft) and in the left visual hemifield (i.e., participants fixated to the right of the shaft). The proposition of a left hemispheric specialization for visuomotor networks predicts that an asymmetrical illusory bias in action between the hands would be independent of handedness (instead, an enhanced illusory bias for the non-preferred hand would point to a dependency on experience) and fixation location. Alternatively, the proposition of lateralized ventral pathways subserving perception predicts that the illusory bias in perception (and perhaps action, see Franz et al. 2009) would be larger for objects presented in the left visual hemifield than in the right visual hemifield, irrespective of the hand that is used or handedness.

## Methods

### Participants

Twelve male right-handed (age range 18–24 years) and twelve male left-handed (17–24 years) undergraduate students from the University of Hong Kong volunteered to participate in the experiment.^1^ Criteria for inclusion were a score of >50 for right-handers and of <50 for left-handers on a culturally modified version of the Edinburgh Handedness Inventory (Oldfield 1971). One right-handed participant was excluded because he showed a substantial and consistent negative illusory bias in the perception task (i.e., *M* = −12.7%). Participants had normal or corrected to normal vision, and gave their written consent prior to the start of the experiment. The local institution’s ethical committee approved the study.

### Materials and apparatus

The shafts consisted of black elongated rods with a width of 12 mm. These rods were made of three layers of thick cardboard and one magnetic strip. The rods were of three different lengths (i.e., 56, 68, and 80 mm). The 56 and 80 mm shafts served as ‘catch trials’ to increase the variation in object lengths, and were not presented within the Müller-Lyer configurations. By contrast, the 68 mm rods were presented both without and within the Müller-Lyer configurations. Black tails-in and tails-out configurations of the Müller-Lyer were printed vertically on separate background sheets of white paper. The length of the tails was 20 mm and their inclination with respect to the shaft was 37°. The participants sat at the table facing a magnetic white board that was on a table top placed at a viewing distance of approximately 60 cm in an inclined orientation (i.e., 10° with respect to the vertical). The rods were presented on the magnetic white board on top of one of the background sheets. The rods were oriented vertically at the body-midline at shoulder height. The hand starting position on the table top was 10 cm from the body-midline to either side, dependent on the hand that was used. This minimized any occlusion of the illusion by the moving hand during grasping. A small red dot indicated the three fixation locations, either centered on the shaft (i.e., central fixation location) or printed on the sheets 10 cm (i.e., 9.6°) to either side of the shaft of the Müller-Lyer configurations. Fixation to the right or left of the shaft ensured that the object was presented in the left or right visual hemifield, projecting to the right or left hemisphere, respectively. A camera placed directly behind the magnetic board was used to control online that the participants fixated as required. The room was illuminated so shadows from the rods and hand were minimized. Two cameras of a Qualisys 3-D motion-capture system recorded small reflective markers attached to the index finger, the thumb and the wrist of both hands at 120 Hz. The reconstructed 3-D coordinates were used to compute hand apertures (using the markers on the index finger and thumb) and reach movement times (using the wrist marker).

### Procedure and design

The participants performed a perception and an action task. In both tasks, the participants closed their eyes between trials and opened them upon verbal instruction by the experimenter. They then fixated the red dot and were instructed to avoid making any further eye movements. In the perception task, participants were instructed to open the hand such that the distance between the thumb and index finger corresponded to the length of the shaft. They were told not to move their hand, but keep it at the starting position. The hand aperture was recorded for 3 s after the participants indicated that they were satisfied with their estimate. In the action task, participants were instructed to make a quick and accurate reach to grasp the rod between the thumb and index finger and lift it. They always started their reach from the starting position while the thumb and index finger made contact. In both tasks, the starting position for the right and left hand trials were to the right and left of the body-midline respectively.

The participants performed the perception and action tasks using their right and left hand in blocks. An ABBA order of blocks was used. Half the participants first performed the perception and action tasks with their right hand; the other half performed both tasks first with their left hand. Additionally, half of the participants started with the perception task, the other half with the action task. The order of hand-use and task was counterbalanced across groups (i.e., left- vs. right-handers). Before each block, participants received 3 practice trials in which they were presented with a 76 mm rod without the Müller-Lyer configuration. Each block of trials consisted of three blocks of fixation location conditions in a counterbalanced order. Within each fixation location condition five stimulus combinations were presented in a randomized order. The 56 mm, 68 mm and 80 mm were each presented twice without the Müller-Lyer configuration and in addition, the 68 mm rod was also presented three times embedded in the tails-in Müller-Lyer configuration and three times within the tails-out Müller-Lyer configuration resulting in 12 trials for each fixation location. Hence, the experiment consisted of 144 trials, 36 trials (i.e., three fixation locations) for both hands and both tasks each.

### Data-analysis and statistics

For the perception task, we calculated the average hand aperture for the first second of the recording, while for the action task the maximal hand aperture and the reach movement time served as the dependent variables. We first controlled for variance that is not attributable to the illusion. To this end, we calculated the difference between the mean hand apertures and reach movement times for the tails-in and tails-out configurations of the Müller-Lyer illusion for each task, hand, and fixation location separately for each participant. This difference was then divided by (respectively) the mean hand aperture and reach movement time for the 68 mm non-illusory configuration. Multiplying by 100% gives the percentages of illusory bias in hand aperture and reach movement time relative to the hand aperture and reach movement time for the non-illusory configurations (for a similar method, see De Grave et al. 2009). Subsequently, the percentage of illusory bias for hand aperture was submitted to a 2 (group; right-handers, left-handers) by 2 (task: perception, action) by 2 (hand: right hand, left hand) by 3(fixation location, left, centre, right) analysis of variance with repeated measures on the last three factors. A separate 2 (group; right-handers, left-handers) by 2 (hand: right hand, left hand) by 3 (fixation location, left, centre, right) analysis of variance with repeated measures on the last two factors was conducted for reach movement time. A Greenhouse-Geisser correction to the degrees of freedom was applied in the case of any violations of sphericity and partial eta-squared (η_p_^2^) values were computed to determine the proportion of total variability attributable to each factor or combination of factors. Post hoc comparisons were performed using Tukey-HSD tests. Two-tailed one sample *t* tests (with test value = 0) were conducted to establish whether the percentage of illusory bias in hand aperture and reach movement time differed from zero for each group, task, hand, and fixation location separately.

## Results

**Fig. 1:**
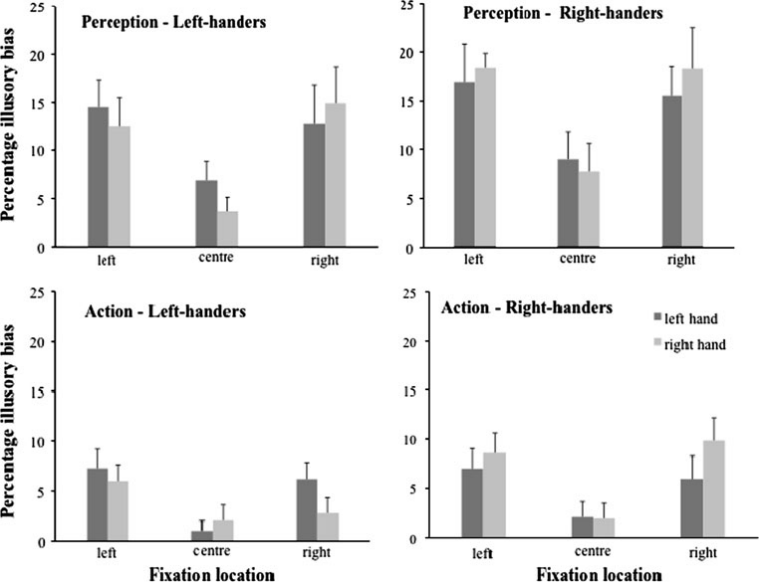
Percentage of illusory bias in hand aperture (and SE) as function of hand used and location fixation. The *top left panel* presents the hand aperture bias for the *left*-handers in the perception task, the *top right panel* presents the *right*-handers in the perception task, the *bottom left panel* presents the *left*-handers in the action task, and the *bottom right panel* presents the *right*-handers in the action task

Figure 1a–d show the percentage of illusory bias in hand apertures in the perception and action tasks for the right-handers and left-handers separately. Inspection of the figures indicates that there were no systematic differences in illusory bias related to the hand that was used or group (i.e., hand-dominance). Rather, the most evident differences in illusory bias seem to be related to task and fixation location.

The analysis of variance confirmed this. There were no significant main effects for group (*F*(1, 21) = 0.44, *p* = 0.52) or hand (*F*(1, 21) = 0.01, *p* = 0.91), nor were there significant interactions involving these factors. Nevertheless, it is noticeable that the group by task interaction almost reached significance (*F*(1, 21) = 3.77, *p* = 0.066, η_p_^2^ = 0.15), suggesting that the right-handers tended to show a larger illusory bias in the perception task than the left-handers (i.e., 14.3 vs. 10.9%), while there was clearly no difference in illusory bias between the groups for the action task (i.e., 5.9 vs. 4.3%). By contrast, significant main effects were revealed for task (*F*(1, 21) = 33.39, *p* < 0.001, η_*p*_^2^ = 0.61) and fixation location (*F*(2, 42) = 26.21, *p* < 0.001, η_*p*_^2^ = 0.52). Post hoc comparisons indicated that the illusory bias was larger in the perception task as compared to the action task and larger when the participants fixated next to the configuration rather than at the configuration. Yet, the later difference was not related to visual hemifield.

One sample *t* tests (with test-value = 0) indicated that for both groups the hand apertures in the action task with gaze fixated at the centre of the configuration were not biased by the illusion (*p*’s > 0.19). Of the remaining *t*-tests (including those for the perception task) all but one were significant (*p*’s < 0.025), indicating that the illusory bias was significantly larger than zero; the exception being left-handers using their right hand to grasp while fixating to the right of the object (*p* = 0.12).

**Fig. 2:**
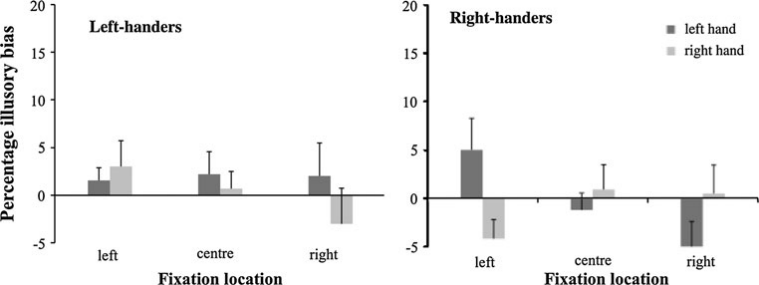
Percentage of illusory bias in reach movement time (and SE) as function of hand used and location fixation. The *left panel* presents the bias in reach movement time bias for the *left*-handers, while the *right panel* presents the bias for the *right*-handers

Figure 2 shows the illusory bias for the movement time of the reach. It shows a relatively small illusory bias that varies around zero^2^ without an immediately illuminating pattern related to hand use, group (i.e., handedness) or fixation location. Nonetheless, the analysis of variance did reveal a significant interaction between group, hand and fixation location (*F*(2, 42) = 3.87, *p* < 0.05, η_p_^2^ = 0.16). Tukey-HSD post hoc comparisons, however, failed to indicate significant differences between means. Finally, the one sample *t* tests (with test-value = 0) showed that the percentages illusory biases for reach movement duration did not exceed zero (*p*’s > 0.06). The lack of a significant illusory bias suggests that differences in reach movement times (if any) are not caused by illusion inducing aspects of the stimulus configurations.

## Discussion

Optical illusions normally impinge conscious perception of objects to a far greater extent than the movements directed to those objects. This larger resistance of action to illusions was also found in the present study. The illusory bias in the perceptual length estimate was clearly larger than in the grasping or reaching movements. Hence, perception seemed more reliant on allocentric or contextual information, whereas movement control was influenced more by egocentric or context-independent information instead. This is consistent with the two-visual systems model, which proposes that perception and action are dissociated in that they are supported by separate neuroanatomical pathways and, among other distinctions, exploit different sources of information (e.g., Milner and Goodale 1995, 2008). Yet, the present study also showed that there is no strict one-to-one mapping between function (i.e., perception and movement control) and information use. That is, movements were not always immune to illusion-inducing allocentric information, suggesting interacting contributions from the two visual systems to movement control. Gonzalez et al. (2006) argued that because of the encapsulation of visuomotor networks for action in the left-hemispheric dorsal stream, the interactive influences of visual perception on movements controlled by the left hemisphere should be reduced relative to movements controlled by the right hemisphere. Accordingly, they reported that right-handed grasps were less susceptible to optic illusions than grasps performed with the left hand (see also Adam et al. 2010; Radoeva et al. 2005). Replication of this asymmetrical illusory bias for the hands in pointing, however, has not always been possible (De Grave et al. 2009; see also Gentilucci et al. 1997). This ambiguity may have resulted from a confound between hand use and visual hemifield. Hence, we used an experimental design to disentangle these factors. Our results showed that the illusory bias was similar for grasping movements with the right and left hand and only occurred for movements that were directed at non-foveated objects irrespective of visual hemifield. For the reaching movements, no significant illusory bias was found. In fact, the difference in illusory bias between grasping and reaching movements corroborates evidence that adaptations to the target size usually manifest more in grasping movements than in reaching movements (e.g., Paulignan et al. 1991). Taken together, we found no evidence that movements performed with the right hand are less prone to the use of allocentric illusion-inducing information than movements with the left hand (see also de Grave et al. 2009). Since the illusion effects emerged irrespective of whether the participants’ used their preferred- or non-preferred hand, also the hypothesis that experience leads towards a stronger reliance on egocentric information (Gonzalez et al. 2008; van der Kamp et al. 2003) was not further substantiated. That said, the precision grips required in the present study were relatively automatized movements, even for the non-preferred hand. This may have reduced the likelihood that an asymmetric illusory bias for the preferred and non-preferred hand occurred (Gonzalez et al. 2008, but see Gonzalez et al. 2006). In sum, we found no indication in our data that the left hand is more susceptible to visual illusions than the right hand, implying that the interactive contributions of the visual systems for perception and action are similar for the two hands. This means that there is no reason—at least for the simple grasping task toward Müller-Lyer targets used the present study—to suggest that movement control is lateralized or more strongly encapsulated in either the left or right hemisphere (Gonzalez et al. 2006; Radoeva et al. 2005).^3^ Obviously, the generality of this claim would be further enhanced if future research replicates the current findings in the context of other geometrical illusions, such as the Ebbinghaus and Ponzo illusions.

Likewise, a greater role for allocentric illusion-inducing information in grasps to the left visual hemifield in comparison to grasps to the right hemifield was expected from the proposition of a right hemispheric specialization of visual perception networks in the ventral stream (Corballis et al. 2000; Milner and Goodale 1995). Indeed, Radoeva et al. (2005, see also Adam et al. 2010; Gentilucci et al. 1997) found, by comparing two groups of patients with unilateral brain damage, that grasps directed to the left visual hemifield resulted in a stronger illusory bias than grasps directed to the right hemifield. By contrast, although in both the perception and the action task of the current study, the illusion unquestionably affected hand apertures when the shaft was not foveated, the magnitude of the biases did not differ between the hemifields (for a similar finding see Bertelson and Morais 1983). This is not to deny right hemispheric dominance in visual perception. Yet, if one takes the right hemisphere to be relatively dominant among left lateralized persons (Corballis et al. 2000), then the tendency towards a smaller illusory bias in the perception task for the left-handed participants compared to right-handed participants might be inconsistent with this conjecture. At the same time, the absence of the handedness effect in the action task underlines the dissociation between visual perception and movement control.

The illusory bias in the grasping movements clearly differed between foveated and non-foveated objects, indicating that participants relied much more on allocentric information when making estimates of or acting upon objects in the peripheral visual field (see also Gentilucci et al. 1997). One explanation for this difference is that the magnitude of the illusion depends on gaze. Instructions to attend to the shaft and ignore the tails of Müller-Lyer illusion have been reported to destroy the illusory bias in perceptual size estimates (Coren and Girgus 1972; Festinger et al. 1968; Predebon 2004, 2006). Furthermore, van Doorn et al. (2009) recently claimed that the differential effects of the Müller-Lyer illusion on action and perception were related to systematic differences in patterns of gaze. They argued that fixation of the different regions of the Müller-Lyer figure is associated with the detection of egocentric (i.e., regions surrounding the shaft) and allocentric information (i.e., regions surrounding the tails). For instance, Van Doorn et al. (2009) found that the more time the participants spent viewing the shaft, the smaller the illusory bias across perception and action tasks. Similarly, in the present study, the fixation of the shaft may have significantly reduced the size of the illusion. In fact, the illusory bias of 6.8% for foveated objects in the perception task is at the lower end of the biases that are typically reported (i.e., 5–18.8%, see Bruno and Franz 2009). It is, however, a significant bias, and hence, an enhanced exploitation of egocentric information due to fixation on the shaft in itself cannot provide a full explanation for the observed difference in the illusory bias for foveated and peripheral objects.

Alternatively, the larger illusory bias for objects in the peripheral visual field may also point to greater reliance on allocentric information for acting upon non-foveated objects. This would run counter to claims that perception and action exploit different information from the peripheral visual fields (Milner and Goodale 1995). The evidence is equivocal, however. Gentilucci et al. (1997) observed a large illusory bias for pointing towards Müller-Lyer figures in the peripheral field, whereas Thompson and Westwood (2007: see also Binsted and Elliott 1999) found no enhanced influence of the illusion on pointing accuracy. These discrepant results may be attributed to differences in experimental procedures and methods. However, we think that they indicate that the difference between central and peripheral fields in itself is not crucial here. Instead, it is to be expected that grasping (or pointing at) non-foveated objects is less automatized and more awkward than grasping foveated objects,^4^ and hence, may be more reliant on allocentric information (see Gonzalez et al. 2008; Van der Kamp et al. 2003).

In conclusion, we examined the hypothesis that the control of movement of the left hand would be more likely to entail the use of allocentric information than movements performed with the right hand. The hypothesis was derived from the idea that within the left hemisphere, which controls the right hand, the visuomotor networks in the dorsal pathway are more strongly dissociated from the visual perception networks in the ventral pathway than in the right hemisphere. We did not find evidence to support this hypothesis; the Müller-Lyer illusion influenced the grasping movements of the right and left hand to the same extent. We also did not find evidence that the illusory effects were different dependent on the visual hemifield toward which the movements are directed. Yet, there was a consistent and pronounced effect of the illusion when grasping objects in the peripheral visual field, suggesting that allocentric information becomes more important in the control of grasping movements directed to nonfoveated objects.

## Footnotes

1 We used male participants only, because earlier work pointed to a gender difference in the magnitude of the bias induced by the Müller-Lyer illusion with male participants showing a larger bias compared to female participants (Rasmjou et al. 1999).

2 Notice that positive values indicate longer movement times for the perceptually smaller object, whereas negative values point to shorter movement times for the perceptually smaller object.

3 It is perhaps important to emphasize that the current behavioural observations by themselves cannot confirm or disprove specific hypotheses regarding the organization of neuroanatomical networks. We are therefore careful to restrict our claims by emphasizing that we were not able to corroborate the neuroanatomical hypothesis.

4 In fact, analysis of variance indicates that the hand apertures for grasp toward nonfoveated non-illusory stimuli are somewhat larger than for the foveated objects, at least for the 56 mm stimuli. This relative overestimation is consistent with the suggestion that grasping peripheral objects is less practiced.
